# Prediction and Comparisons of Turpentine Content in Slash Pine at Different Slope Positions Using Near-Infrared Spectroscopy

**DOI:** 10.3390/plants11070914

**Published:** 2022-03-29

**Authors:** Qifu Luan, Shu Diao, Honggang Sun, Xianyin Ding, Jingmin Jiang

**Affiliations:** 1 Research Institute of Subtropical Forestry, Chinese Academy of Forestry, Hangzhou 311400, China; qifu.luan@caf.ac.cn (Q.L.); diaoshu0802@163.com (S.D.); 10020031215@sina.com (H.S.); xianyinding@gmail.com (X.D.); 2 National Forestry and Grassland Engineering Technology Research Center of Exotic Pine Cultivation, Hangzhou 311400, China

**Keywords:** model calibration, turpentine, NIR spectroscopy, slope position, slash pine

## Abstract

Pine resin is one of the best known and most exploited non-wood products. Resin is a complex mixture of terpenes produced by specialized cells that are dedicated to tree defense. Chemical defenses are plastic properties, and concentrations of chemical defenses can be adjusted based on environmental factors, such as resource availability. The slope orientation (south/sunny or north/shady) and the altitude of the plantation site have significant effects on the soil nutrient and the plant performance, whereas little is known about how the slope affects the pine resin yield and its components. In total, 1180 slash pines in 18 plots at different slope positions were established to determine the effects on the α- and β-pinene content and resin production of the slash pine. The near-infrared spectroscopy (NIR) technique was developed to rapidly and economically predict the turpentine content for each sample. The results showed that the best partial least squares regression (PLS) models for α- and β-pinene content prediction were established via the combined treatment of multiplicative scatter correction–significant multivariate correlation (MSC–sMC). The prediction models based on sMC spectra for α- and β-pinene content have an R^2^ of 0.82 and 0.85 and an RMSE of 0.96 and 0.82, respectively, and they were successfully implemented in turpentine prediction in this research. The results also showed that a barren slope position (especially mid-slope) could improve the α-pinene and β-pinene content and resin productivity of slash pine, and the β-pinene content in the resin had more variances in this research.

## 1. Introduction

Resins contained in many tree species, such as pines (which produce pine rosin and turpentine), are a renewable raw material for products such as high-grade perfumes, adhesives, and inks [1,2,3]. The important economic application value of resin should be reflected in the relative content of its main components, as production often requires certain components rather than the entire resin [4]. The main components of turpentine are α-pinene and β-pinene, which especially have a wide range of therapeutic potential [5] and have huge impact on the value of the resin. The quick and economical identification and analysis of the monoterpene content in different resins is significant for the production and processing of related industrial raw materials [6,7].

Chemical composition analysis is usually performed using a gas chromatography–mass spectrometry (GC–MS) system. Stable and efficient GC or GC–MS analysis methods has been established for the chemical composition analysis of pine resin, which can obtain the relative content of main components [8,9]. Recently, the methods based on the GC or GC–MS for verification and analysis of volatile essential oil composition (mainly including the α and β-pinene) were developed [10,11]. However, in addition to the expensive GC–MS spectrometry instrument, the GC–MS analysis method has high requirements for the experimental level and analytical ability of the operator. Moreover, it is expensive and time-consuming to analyze each sample, and the identification and analysis of off-line data can only be completed by professional technicians with a chemical background [12].

Therefore, rapid and nondestructive vibrational spectroscopy methods for the rapid determination of oleoresin composition are valuable. The near infrared (NIR) spectroscopy region extends from 780 to 2500 nm, in which the spectra may be characterized by the assignment of the absorption bands to overtones and combinations of fundamental vibrations associated with C-H, O-H, and N-H bonds [13]. The content of many oil compositions, such as α- and β-pinene from plant resources or the content of resin and rubber, were estimated by NIR spectroscopy methods [14,15]. Vibrational spectroscopy methods for oleoresin composition analysis are promising. However, few studies have reported on the content of pine oleoresin estimated by NIR spectroscopy methods. Partial least squares (PLS) regression, a quick, efficient, and optimal regression method for the construction of prediction models based on vibrational spectroscopy, is a widely used chemometric method [16]. It is worth noting that substantial spectral data will contain redundant and complicated information. Therefore, to establish a moderately practical model, it is necessary to preprocess the collected spectral data [17]. Preprocessing NIR spectral data has since become a crucial step in chemometric modeling. Similarly, variable selection is also a critical step in spectral analysis, which can select the most relevant spectral band to improve the model’s overall performance [18].

Resin terpene synthesis in conifers is influenced not only by genetic factors but also by climatic and environmental factors such as soil fertility, stand dominance, tree growth, age, season, temperature, wounding, and disturbance [2,19]. According to Sampedro et al. [20], chemical defenses are plastic properties, and concentrations of chemical defenses can be adjusted on the basis of the environment and by the interaction effect of genotypes on environmental conditions such as resource availability. Genetic effects influencing the content of pine oleoresin components were studied by Zhang et al. [7], Lai et al. [4], and Yi et al. [21]. Individual heritability was moderate for resin yield and moderate to high for monoterpene components at different sites. A significant site effect for most of the studied properties was observed with the joint analysis of all trials. The estimates of type-B genetic correlations showed that the genotype-by-environment (G × E) interaction had a relatively strong influence on resin yield and most of the resin chemical components [4]. The compounds and their content of the essential oil from the needles or twigs of pine species of different geographical regions are also varied because of the environmental differences during different seasons [22,23].

Pine plantations are primarily located in the low hills of subtropical areas in southern China [24]. A lower content of organic matter and microorganisms and lower activity of the enzymes related to microbiological activity in the soil on the north-facing slope were observed [25]. The research showed that trees on sunny slopes had higher growth than the trees on shady slopes, whereas trees in mid-slope positions with shallower soils and high sodicity showed the lowest aboveground biomass, stem biomass, and total height yield [26]. Soil-available water was the primary factor for plant productivity. All growth parameters in Aleppo pine trees obtained on valley bottoms were significantly higher than all aspect slope position combinations due to the accumulation of runoff and deposition from the upper to middle and finally to lower slopes [27].

All studies have shown that the slope direction and position at one site have significant effects on soil nutrients and plant performance [26,27,28,29]. However, little is known about how the slope affects the pine resin yield and its components. China’s output of pine resin has reached more than 60% of the world’s output, and pine resin is one of the most important parts of forestry, with an annual output value of more than 8 billion yuan in recent years. Slash pine (*Pinus elliottii* Engelm var. *elliottii*) is one of the leading tree species for resin tapping in China [30]. Thus, it is important to know how the slope direction and position affect the pine resin yield and the components for field management and resin production.

Therefore, this paper aims (1) to derive a technique to reliably estimate and predict the content of α- and β-pinene in the resin of slash pine using NIR technology and (2) to determine the effects of slash pine and resin production at different slope positions on the α- and β-pinene content using the NIR-based technique.

## 2. Materials and Methods

### 2.1. Sites and Plots

The study sites were located in the western suburbs of Hangzhou city, China (30°3′ N, 119°57′ E). The site belongs to subtropical low hill area with an east to west orientation. Slash pine plantations were established on the two slopes of the hill in January 1999 with a spacing of 3 m between rows and 2 m between trees within a row. In total, 1180 trees of 18 plots at different positions (P) and directions of slope (SO) were established in the spring of 2019, and the length and width for each plot was 25 m (Table 1). Each plot had an average of 65 trees, ranging from 50 to 85.

### 2.2. Growth Measurements and Resin Collection

**Figure 1:**
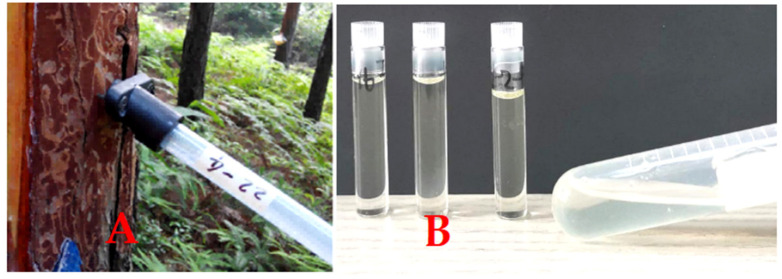
Resin collection (**A**) and NIR scan (**B**) device.

The tree height and DBH (diameter at breast height) were measured in the spring of 2019. The collection of oleoresin production was done in August 2019 using the special tube (Figure 1A) method of Zhang et al. [7]. The resin collected in the 1180 plastic tubes was measured for resin productivity (RP). The resin in plastic tubes (Figure 1A) was then transferred to glass tubes (Figure 1B) for NIR spectral data collection as soon as possible.

### 2.3. Collection of NIR Spectral Data

The NIR spectral data from all 1180 resin samples were collected by using near-infrared spectroscopy (XDS™ NIR Rapid Liquid Analyzer, FOSS). For each scan, the resin sample was placed in the glass tube (Figure 1B) dedicated to the Foss spectrometer, and scanned spectra were averaged after 20 scans per sample, whose values ranged from 400 nm to 2500 nm with a 2 nm resolution. A total of 143 resin samples in the glass tubes were selected randomly for GC–MS analysis after collection of the NIR data.

### 2.4. Resin Analysis by GC–MS

The GC–MS method was carried out for the 143 samples using an HP6890GC/5975B gas chromatograph and the mass spectrometry (Agilent 5975B, Santa Clara, CA, USA) for qualitative and quantitative analysis of oleoresin composition with the chromatographic condition as follows: GC: 0.05 g of oleoresin was dissolved in 0.5 mL of ethyl alcohol containing 50 µL tetramethylammonium hydroxide and analyzed by using a DB-5MS silica capillary column (60 m × 0.25 mm internal diameter, 0.25 µM film thickness). The initial column temperature was 60 °C for 2 min, increased to 80 °C for 5 min, and reaching a maximum of 280 °C at a rate of 2 °C per min for 5 min. Injector temperature was 260 °C. The injection volume was 1 µL with a 1/50 split ratio. The carrier gas was helium. EI-MS: the electron energy was 70 eV. The connection parts and ion source temperatures were 250° and 230 °C, respectively. The mass scan range was 30 to 600 *m/z* along with solvent delay for 3 min.

Resin compositions were identified by matching experimental fragmentation patterns in mass spectra with the NIST08 database through the data processing system of Agilent Chem Station and then comparing with the relevant literature [9]. The relative content of each component determined by peak area normalization is expressed as a percentage of the total amount of components.

### 2.5. Preprocessing and Variable Selection of NIR

To reduce bias from physical factors and irrelevant variables on the establishment of a stable and reliable model [31], four preprocessing methods, namely, standard normal variate (SNV), multiplicative scatter correction (MSC), derivative method (DM), and block normal (BN), were combined with PLS [32]. The calibration set (n = 100 samples) was used to develop a calibrated model, and the separate validation set (n = 43 samples) was reserved to assess and evaluate the prediction performance of the developed model. Two indicators of internal cross-validation, the correlation coefficient (R^2^) and root mean square error (RMSE), were used to assess model robustness. For that, the closer R^2^ is to 1 and RMSE is to 0, the better the prediction ability of the model is [33]. We used sMC (significant multivariate correlation) [34] as a variable selection method to determine the best PLS model performance with fewer spectral variables.

### 2.6. Software Tools

All NIR data analysis and model building were implemented in Unscrambler (v10.2, CAMO, Software AS, Norway). R software (V4.0.5) was used in the basic analysis and for drawing the plots [35].

## 3. Results

### 3.1. Establishment of α- and β-Pinene Content Models Based on PLS

**Figure 2:**
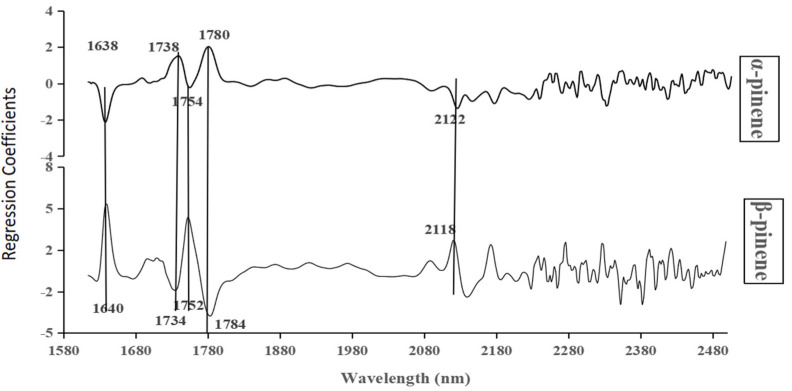
The range of characteristic spectra based on sMC for α-pinene and β-pinene and the regression coefficients of the spectra to the pinene content. The numbers near the curves are the wavelengths of the corresponding curve peaks.

Four spectral preprocessing methods were used for model calibration (n = 100 samples), and the results showed that the MSC method with a full or characteristic NIR spectrum was best for model establishment. For example, the MSC preprocessing method produced the most accurate α-pinene content prediction model, with R2 and RMSE values for the calibration set of 0.89 and 0.74, respectively (Table 2). To improve the model, a characteristic spectral band (based on sMC) was selected, which showed that many significant regression coefficients for α-pinene and β-pinene at wavelengths of 1638, 1640, 1734, 1738, 1752, 1754, 1780, 1784, 2118, and 2122 nm were opposite in sign (Figure 2). For example, the regression coefficients were −2.12 (1638 nm) and 4.81 (1640 nm) for α- and β-pinene, respectively. The characteristic spectral ranges of α-pinene and β-pinene were under 50% of the full spectra (400 nm–2500 nm). The results of the prediction and calibration models with the full NIR spectrum and characteristic spectra are shown in Table 2. The calibration and prediction models both showed that there were superior R^2^ and minor RMSE values in model establishment with characteristic spectra than with full spectra (Table 2).

### 3.2. Separate Validation to Evaluate the Prediction Performance

**Figure 3:**
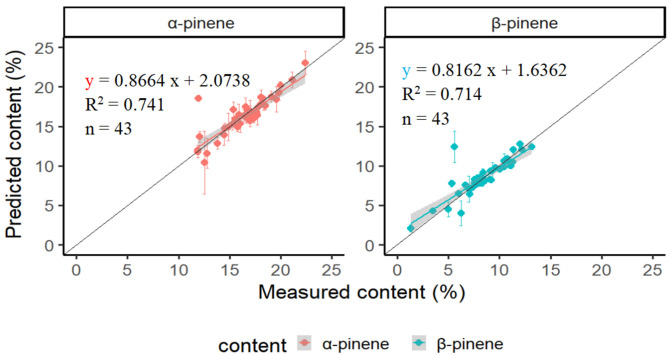
The α- and β-pinene content for the separate validation samples predicted by the PLS model and their relationship between measured and predicted.

The separate validation set (n = 43 samples) was reserved to evaluate the prediction performance of the developed model. The prediction results are shown in Table 3 and Figure 3. The average α-pinene content measured by GC–MS (reference) and the predicted model (predicted) was 16.55% and 16.41%, respectively, and the deviation for the predicted value was 0.91. The average β-pinene content was 8.34% and 8.44% for the reference and predicted values, respectively, with a lower deviation of 0.49 for the predicted value. The linear equation for the predicted (y) and reference (x) values is listed in Figure 3, which shows a higher determination coefficient (R^2^) of 0.741 and 0.714 for α-pinene and β-pinene, respectively.

### 3.3. Comparisons of α-Pinene and β-Pinene Content Percentages in Slash Pine at Different Positions Using the PLS Model

**Figure 4:**
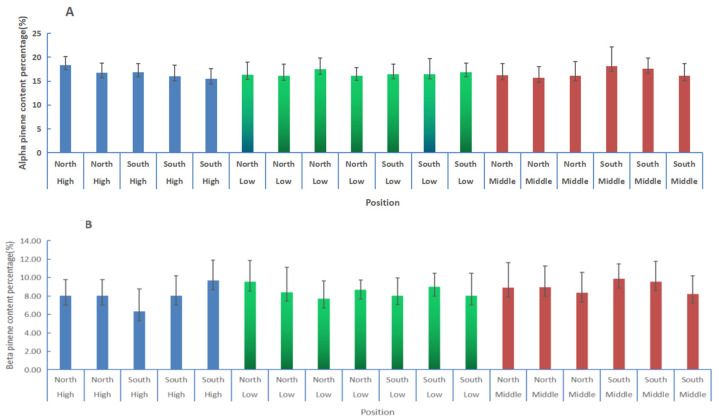

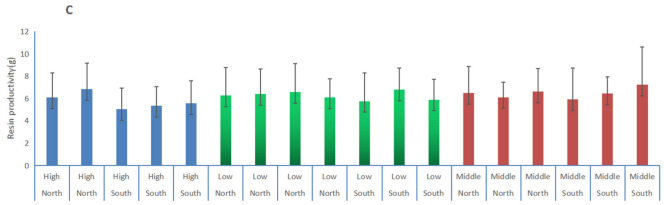
The α-Pinene (**A**) and β-pinene (**B**) contents and resin productivity (**C**) of the trees in each plot.

**Figure 5:**
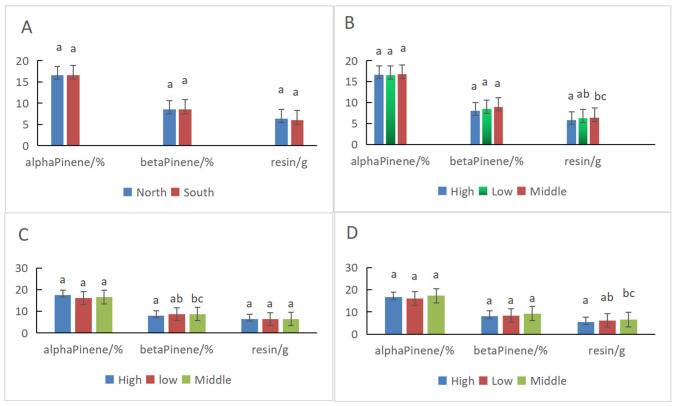
The α-Pinene and β-pinene contents and resin productivity for the trees in different plots and their comparison ((**A**): North vs. South slope; (**B**): Elevation; (**C**): North slope; (**D**): South slope). Duncan’s multiple-range test; means with the same letter are not significant (*p <* 0.05).

The characteristic spectra model was used to predict the α-pinene and β-pinene contents of slash pines in the 18 plots established at different positions of a low hill plantation (Figure 4A,B). The resin productivity (Figure 4C) of each tree in the plots was analyzed. The differences in the resin components are shown in Figure 5 and Table 4.

There were no differences on the α-pinene content, β-pinene content, and resin productivity for the trees between the north and south slopes (Figure 5A and Table 4). The mean α-pinene content on the northern and southern slopes was 16.61% and 16.62%, respectively, which are almost the same. Additionally, the β-pinene content was 8.51% and 8.53%, respectively. The resin productivity in the north was 6.00 g, which was slightly higher than the 6.40 g on the south slope.

There were no differences in the α-pinene and β-pinene content for the trees between the different elevations combined with the results of the south and north slopes, whereas the resin productivity at the middle elevation was significantly higher than that at the high elevation (Figure 5B and Table 4). Moreover, the α-pinene and β-pinene content and resin productivity at the middle elevation were higher than those at the low and high elevations.

The elevation of the north slope had significant effects on the β-pinene content. The β-pinene content at the middle elevation on the north slope was significantly higher than that at the high elevation, whereas there were no differences in the α-pinene content and resin productivity at different elevations on the north slope (Figure 5C and Table 4). There were significant differences in resin productivity between the middle and high elevations of the south slope (Figure 5D and Table 4). The elevation on the south slope had no significant effects on the α- and β-pinene content. Again, the α-pinene and β-pinene content and resin productivity at the middle elevation of the south slope were higher than those at low and high elevations.

In total, the α-pinene content was not significantly different at different elevations and slopes. The β-pinene content was significantly affected by the elevation of the northern slope, and the resin productivity was significantly affected by the elevation of the southern slope. The mean α- and β-pinene content and resin productivity were not affected significantly by slope orientation.

## 4. Discussion

The purpose of this study was to reveal the applicability of NIR for the prediction of α- and β-pinene content in the resin of slash pine and to determine how the slope orientation and altitude position affect the α- and β-pinene content in the resin of *Pinus elliottii* using the NIR-based technique. The resin production of each plot was also considered because of its significant difference.

In our study, the relationship between α- or β-pinene and NIR spectra was explored using selected spectral preprocessing methods and characteristic variables. Comparing the results in Table 2, The R^2^ values were slightly lower than those reported for predicting the pinene or resin content (R^2^ was approximately 0.90) in pepper (*Piper nigrum* L.) [14] and guayule (*Parthenium argentatum*) [15]. The reason for this disparity may be the complexity of the measured materials. The essential oils distilled from the peppers were mostly volatile matter, including approximately ten components, and the resin from the guayule was measured as one compound for prediction. There are more than 40 components [8] in the sticky resin collected from slash pine in this research, which could have introduced relatively more irrelevant information in the collected spectral data, which reduced the modeling accuracy of α- and β-pinene content.

It is necessary to select effective spectral information to improve the accuracy of a fitted model [36]. Moreover, smaller wavelengths are feasible for using smaller and lighter portable spectrometers in field applications [37]. In our study (Table 2 and Figure 2), many significant regression coefficients for α-pinene and β-pinene at the reduced characteristic spectral band (based on sMC) were opposite in sign because the two chemicals approximate the chemical structure with the same molecular weight and elemental composition (C10H6). Their content had a significant negative correlation in slash pine [7].

Pines secrete resins for their protective benefit in response to injury. Conifers have evolved complex oleoresin terpene defenses against herbivores and pathogens [38]. The rate and amount of resin flowing from wounds, the pressure and composition of resin, and the size and number of resin ducts contribute to conifer tree resistance against abiotic and biotic injury. Resin pressure within the ducts and the flow of resin from wounds are directly affected by environmental variables, such as temperature and availability of water [39]. Thus, the amount of resin is not always significantly related to the biomass of trees because it responds to injury and environmental variables [11,38,39], especially adverse situations, as noted above. That is why the resin productivity of the trees on the south slope was slightly lower than that on the north slope, but the tree height and DBH showed the opposite results in this research. There were no significant differences in the α-pinene and β-pinene content and resin productivity between the trees on the north and south slopes (Table 1 and Table 4, Figure 5A), which showed that the average difference in the environments between the two slopes was not significant for resin production or components. In contrast, the differences between different regions [22,23] were significant for the constituents of essential oil.

We also found that the α-pinene content was not significantly different at any position, whereas the β-pinene content had more variance (Table 4, Figure 5), which showed that α-pinene was more stable than β-pinene in the resin of slash pine. Lai et al. [4] also found a significant site effect on β-pinene (*p* < 0.001) and a weak site effect on α-pinene (*p* = 0.404). The significant negative correlation between α- and β-pinene content in the resin of slash pine [4,7] was the typical characteristic, and the reason would be correlated to the synthetic route and biological functions of the two monoterpenes. The C_5_ monomeric precursors in plants were finally converted into monoterpenes (C_10_) by the action of shared terpene synthases [38,40], which means that more α-pinene was synthesized and less β-pinene was produced.

In this research we mostly discussed the α- and β-pinene content in the resin tapping from the stem of the slash pine. The content of α- and β-pinene in the essential oil distilled from the needles or twigs of other pine species such as *Pinus nigra* [22,23] or *Pinus sylvestris* [11] have different rules. For example, the α- and β-pinene content in the essential oil distilled from needles of *Pinus nigra* in Denizli, Turkey [23] was 4.51–49.63% and 1.42–13.07%, respectively, which showed that the α-pinene content in the essential oil had more variance.

## 5. Conclusions

Our results have shown that we can use NIR spectroscopy to quickly and accurately predict the α- and β-pinene content in resin. The most important wavelength regions were found by the sMC variable selection method, which showed that many significant regression coefficients for α-pinene and β-pinene at specific wavelengths were opposite in sign. The prediction models were successfully implemented in turpentine prediction research as a reliable and economical method. The results also showed that a barren slope position (especially mid-slope) could improve the α-pinene and β-pinene contents and resin productivity of slash pine, and the β-pinene content in the resin had more variances in this research.

## Figures and Tables

**Table 1:** The position and tree information for each plot.

Slope Orientation	Slope Angle	Plot No.	Altitude	Position	Average of Tree Height	Average of Diameter at Breast Height
South (Sunny)	20°	10, 13, 16	10 m	Low	9.8 m	12.1 cm
		11, 14, 17	30 m	Middle	8.9 m	10.8 cm
		12, 15, 18	50 m	High	9.1 m	11.3 cm
North (Shady)	21°	3, 4, 5, 6	10 m	Low	9.7 m	11.9 cm
		7, 8, 9	30 m	Middle	8.7 m	10.5 cm
		1, 2	50 m	High	8.8 m	11.1 cm

**Table 2:** The indicators of internal cross-validation, the correlation coefficient (R^2^) and root mean square error (RMSE) for the calibration and prediction model of α- and β-pinene content based on full and characteristic spectra.

Model		Calibration		Prediction	
	Spectra	R^2^	RMSE	R^2^	RMSE
α-pinene content	Full Spectra (400 nm–2500 nm)	0.8927	0.7435	0.8157	0.9850
	Characteristic spectra (selected by sMC)	0.8953	0.7239	0.8203	0.9579
β-pinene content	Full Spectra (400 nm–2500 nm)	0.9314	0.5405	0.8389	0.8349
	Characteristic spectra (selected by sMC)	0.9418	0.4877	0.8464	0.8167

**Table 3:** The α- and β-pinene content of the separate validation samples predicted by the corresponding PLS model.

Samples	α-Pinene Content (%)			β-Pinene Content (%)		
	Reference	Predicted	Deviation	Reference	Predicted	Deviation
PEE101	19.98	20.14	0.52	3.50	4.31	0.32
PEE102	17.29	16.42	0.72	10.06	9.57	0.32
PEE103	17.80	17.40	0.50	7.55	8.30	0.27
PEE104	12.81	11.58	1.91	7.03	7.07	1.62
PEE105	21.12	20.86	0.99	6.24	3.98	1.58
PEE106	17.38	15.99	0.54	7.77	7.63	0.29
PEE107	18.52	17.61	0.47	7.09	6.45	0.39
PEE108	19.07	18.78	0.69	7.42	7.30	0.39
PEE109	18.05	17.82	0.45	7.03	7.06	0.30
PEE110	11.90	12.00	0.62	12.22	12.04	0.35
PEE111	14.53	14.77	0.61	10.74	10.91	0.32
PEE112	17.67	16.76	0.68	7.88	8.46	0.27
PEE113	16.63	16.13	0.70	8.31	7.80	0.35
PEE114	19.82	19.24	0.93	5.60	12.41	1.99
PEE115	12.09	13.66	0.65	11.39	12.04	0.39
PEE116	16.81	16.96	0.50	9.06	8.25	0.29
PEE117	17.16	16.50	0.50	7.91	7.77	0.31
PEE118	14.87	14.91	1.74	8.40	9.13	0.49
PEE119	11.96	18.57	0.42	5.33	7.76	0.41
PEE120	15.78	14.87	0.56	8.30	8.08	0.30
PEE121	17.42	16.70	0.57	8.16	8.53	0.31
PEE122	15.38	17.13	0.96	9.35	9.42	0.98
PEE123	16.55	17.48	1.14	7.57	7.84	0.31
PEE124	18.32	17.72	0.68	8.60	8.10	0.30
PEE125	16.72	16.31	0.69	7.86	8.20	0.30
PEE126	16.41	16.34	0.74	6.65	7.55	0.37
PEE127	18.23	18.52	0.46	8.01	7.79	0.31
PEE128	16.05	15.31	1.05	10.46	9.85	0.30
PEE129	17.01	16.74	0.84	9.18	8.19	0.30
PEE130	18.09	18.71	0.87	6.03	6.47	0.31
PEE131	15.28	15.31	0.78	11.04	10.02	0.32
PEE132	16.97	16.68	0.67	11.15	9.98	0.31
PEE133	13.81	12.83	0.73	13.18	12.39	0.34
PEE134	12.53	10.39	3.99	10.48	10.61	0.97
PEE135	17.01	15.71	0.74	11.32	10.51	0.32
PEE136	19.58	18.42	1.61	7.07	6.96	0.62
PEE137	11.89	11.75	0.67	9.65	9.75	0.33
PEE138	15.89	16.45	1.36	9.11	9.34	0.33
PEE139	15.54	15.87	0.46	8.01	7.77	0.37
PEE140	17.74	16.43	1.26	5.02	4.51	0.91
PEE141	14.45	13.89	1.30	12.04	12.73	0.39
PEE142	22.41	23.05	1.44	1.34	2.09	0.46
PEE143	17.02	16.98	1.31	8.53	8.18	0.50
Mean	16.55	16.41	0.91	8.34	8.44	0.49

**Table 4:** The α-pinene and β-pinene contents and resin productivity for the trees in different plots and their multiple comparisons *.

Orientation	Position	Alpha Pinene/%	STDEV	Beta Pinene/%	STDEV	Resin /g	STDEV
North	All	16.61 a	2.30	8.51 a	2.09	6.40 a	2.14
South		16.62 a	2.48	8.53 a	2.28	6.00 a	2.18
All	High	16.68 a	2.02	8.02 a	2.07	5.78 a	2.04
	Low	16.55 a	2.33	8.49 a	1.99	6.27 ab	2.17
	Middle	16.72 a	2.83	9.00 a	2.46	6.47 bc	2.23
North	High	17.54 a	1.94	8.03 a	2.28	6.48 a	2.56
	Low	16.18 a	1.74	8.59 ab	2.01	6.35 a	2.42
	Middle	16.57 a	2.28	8.74 bc	2.23	6.42 a	1.93
South	High	16.59 a	2.08	8.01 a	2.29	5.32 a	1.88
	Low	16.02 a	2.40	8.36 a	1.96	6.16 ab	2.09
	Middle	17.26 a	2.96	9.23 a	2.59	6.53 bc	2.57

* Duncan’s multiple-range test, means with the same letter are not significant (*p* < 0.05).

## Data Availability

Not applicable.
